# Radiomics based on habitat analysis in predicting parametrial invasion of early stage cervical cancer

**DOI:** 10.3389/fonc.2026.1694347

**Published:** 2026-01-27

**Authors:** Chongshuang Yang, Man Li, Changfu Yang, Peng Jiang, Changyi Yang, Junfeng Dai, Bing Chen, Wei Wang, Zhihong Qin, Tianliang Shi, Xin Yi, Zhihai Jin

**Affiliations:** 1 Department of Radiology, Tongren People’s Hospital, Tongren, Guizhou, China; 2 Department of Radiology, Faculty of Medicine and Health Sciences, Universiti Putra Malaysia, Serdang, Selangor, Malaysia; 3 Department of Research and Development, Shanghai United Imaging Intelligence Co., Ltd., Shanghai, China; 4 Department of Internal Medicine, Faculty of Medicine and Health Sciences, Universiti Putra Malaysia, Serdang, Selangor, Malaysia; 5 Department of Orthopaedic, Handan First Hospital, Handan, Hebei, China; 6 Department of Community Health, Faculty of Medicine and Health Sciences, University Putra Malaysia, Serdang, Selangor, Malaysia

**Keywords:** cervical cancer, habitat analysis, magnetic resonance imaging, parametrial invasion, radiomics

## Abstract

**Objective:**

To evaluate radiomics based on habitat analysis for preoperatively predicting parametrial invasion (PMI) in clinically early-stage cervical cancer (CC).

**Methods:**

This retrospective study included 110 consecutive patients clinically staged as IB-IIA before treatment. Patients were randomly divided into the training and testing cohorts in an 8:2 ratio. Regions of interest were manually delineated on T2-weighted images, which were then segmented into sub-regions using a k-means clustering algorithm based on voxel intensity and entropy values. Radiomic features were then extracted from both the whole tumor and each sub-region. Feature selection was performed using correlation analysis, recursive feature elimination, and the least absolute shrinkage and selection operator method. Subsequently, models were constructed based on valid radiomics features extracted from the whole tumor and from each sub-region. The diagnostic accuracy of the models was evaluated using receiver operating characteristic analysis. The area under the curve (AUC) was compared descriptively, and the analysis was supplemented by net reclassification improvement and comprehensive discrimination improvement measures.

**Results:**

Tumors were divided into three sub-regions (habitat 1-3). A total of 2260 and 1890 radiomics features were extracted from whole tumor and each habitat, respectively. After selection, 10, 10, 7 and 9 valid features were selected from whole tumor and habitats 1-3, respectively. All models had good classification performance for positive and negative PMI in the training and testing cohorts, with an AUC ranging from 0.777 to 1.00 in the training cohort and from 0.750 to 0.850 in the testing cohort. In addition, the diagnostic performance of habitat 3 was higher than that of the whole tumor, habitat 1, habitat 2 models in the training and testing cohorts, and the difference was statistically significant (p<0.05). The sensitivity, specificity, and AUC (95% confidence interval) of habitat 3 model in the training and testing cohorts were 97.9%, 100%, 1.00 (0.999–1.00) and 75.0%, 100%, 0.850 (0.649–1.00), respectively.

**Conclusion:**

Radiomics based on habitat analysis effectively predicts PMI in early-stage CC, with diagnostic performance superior to that of traditional whole-tumor radiomics. This approach provides a promising method for preoperative prediction of PMI in CC and aids clinicians and patients in treatment decisions.

## 1 Introduction

Cervical cancer (CC) continues to be the most prevalent malignant tumor affecting the female reproductive system globally, posing a significant threat to women’s health (1). Accurate preoperative clinical staging is crucial for determining optimal treatment strategies, with parametrial invasion (PMI) being a key factor influencing stage classification. According to the International Federation of Gynecology and Obstetrics (FIGO) staging system, CC involving two-thirds of the vagina without PMI is classified as stage IIa and is primarily treated with radical surgery, whereas the presence of PMI upgrades the disease to stage IIb, for which concurrent chemoradiotherapy is the preferred treatment modality (2). Therefore, reliable pre-treatment evaluation of PMI is of great clinical importance for guiding individualized therapy.

Magnetic resonance imaging (MRI), particularly T2-weighted imaging (T2WI), is currently the most used non-invasive tool for assessing PMI (3). Radiologists often determine PMI status by observing disruption of the mid-stromal ring or loss of cervical hypo-intensity on T2WI (4). However, due to tumor compression, peritumoral edema, or inflammatory changes, conventional MRI assessments are prone to false-positive results (5, 6), limiting their diagnostic accuracy. Even some functional MRI techniques, such as diffusion-weighted imaging (7), dynamic contrast-enhanced MRI, intravoxel incoherent motion diffusion-weighted imaging (8), and amide proton transfer imaging (9), have not achieved satisfactory diagnostic accuracy in assessing PMI.

Radiomics extracts numerous quantitative features from medical images, revealing information that is often imperceptible to the naked eye (10). Compared to traditional imaging techniques, radiomics has demonstrated significant advantages in tumor characterization (11) and treatment prediction (12). In recent years, several studies have investigated the potential of MRI-based radiomics for preoperative prediction of PMI in CC. Early work established radiomics nomograms based on T2WI (13), demonstrating higher diagnostic accuracy than conventional MRI assessment. Subsequent studies expanded feature extraction to both intratumoral and peritumoral regions (14) and integrated multiparametric MRI sequences (15) to enhance model stability and predictive power.

However, conventional radiomics typically treats the entire tumor as a homogeneous entity during feature extraction, failing to fully capture the intrinsic heterogeneity within the tumor (16). This limitation can compromise the predictive accuracy and robustness of radiomics models. To address this issue, habitat analysis based on radiomics has been introduced. Habitat analysis enables a more detailed characterization of tumor biological behavior by partitioning tumors into distinct subregions and extracting features from each (17). Recent studies have shown that habitat analysis outperforms traditional radiomics approaches in predicting various pathological features of tumors (18). Nevertheless, to date, no studies have explored the application of habitat analysis based on radiomics in predicting PMI in CC.

## 2 Materials and methods

### 2.1 Patient collection and image acquisition

This retrospective study analyzed the preoperative MRI images and clinicopathological data of CC patients who were clinically staged as FIGO IB–IIA based on preoperative evaluation and underwent radical hysterectomy at our hospital between March 2020 and October 2024. A total of 357 patients with suspected CC were initially screened. Among them, 43 patients were excluded because the diagnosis was not CC, 118 patients had received radiotherapy or chemotherapy, and 53 patients had incomplete treatment information. Of the remaining 143 patients who underwent surgery, 21 patients did not have preoperative MRI scans, 8 patients lacked pathologically confirmed PMI status, and 4 patients had images that could not be retrieved. After applying these exclusion criteria, a total of 110 patients were included in the final analysis.

The criteria for inclusion were as follows: (1) patients were pathologically diagnosed with CC, were clinically staged as FIGO IB–IIA before treatment, and their PMI status (positive or negative) was confirmed by postoperative pathology; (2) no history of radiotherapy or chemotherapy prior to MRI scanning; and (3) MRI examinations performed according to standard clinical protocols. The exclusion criteria were: (1) MRI images of insufficient quality for tumor segmentation; and (2) an interval exceeding two weeks between MRI scanning and surgery.

All patients were scanned using a 3.0 T MRI scanner. T2WI and contrast-enhanced T1 weighted imaging (CE-T1WI) images were acquired with the following parameters: T2WI, repetition time (TR)/echo time (TE) = 2500/70 ms, field of view (FOV) = 23 × 36 cm, matrix size = 264 × 320, slice thickness/gap = 5/1 mm. CE-T1WI, TR/TE = 485/8 ms, FOV = 32 × 28 cm, matrix = 300×377, layer thickness/layer spacing = 5/1 mm.

**Figure 1:**
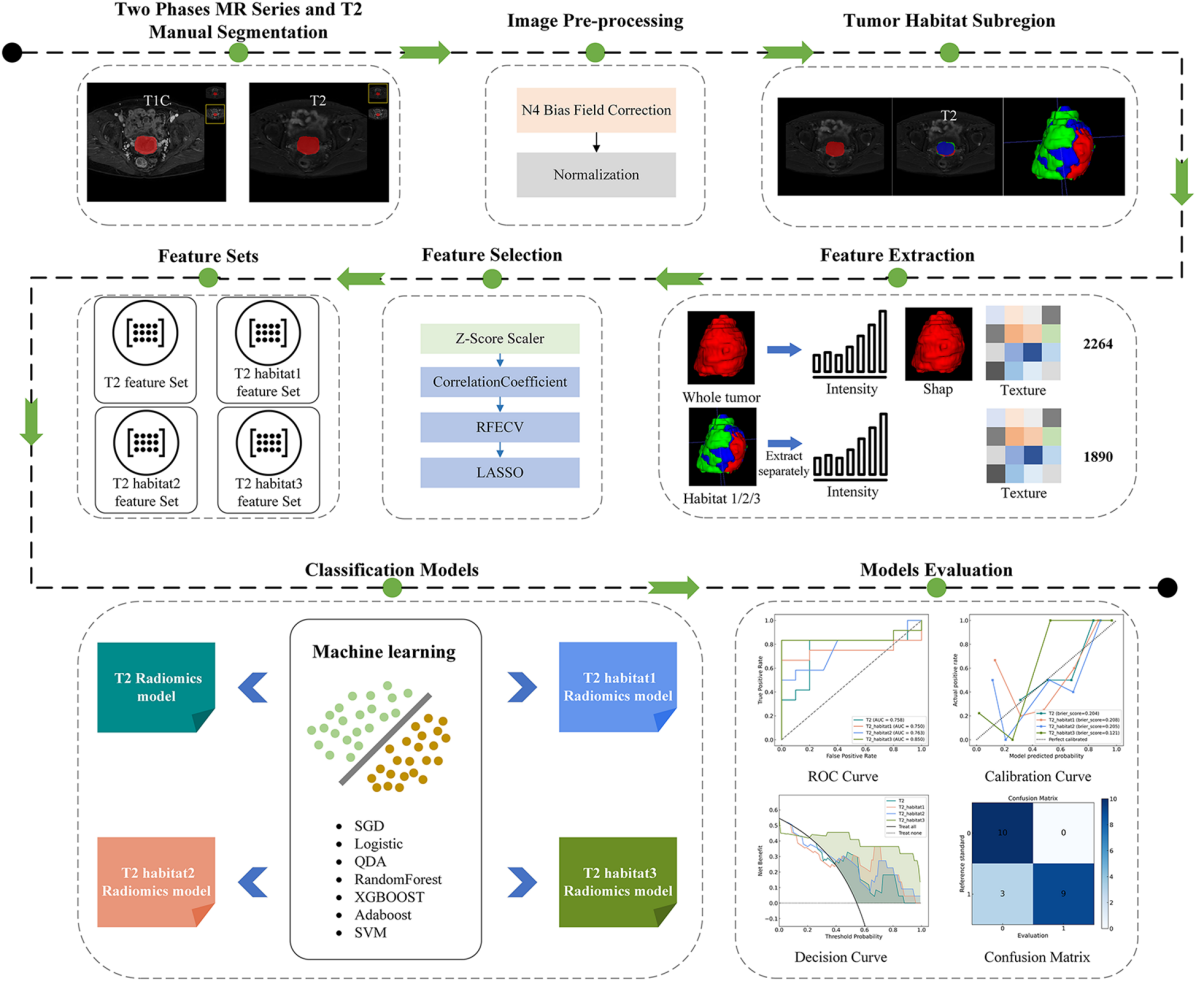
Workflow of this study. MR images were manually segmented and preprocessed (N4 bias correction and normalization), followed by tumor subregion (habitat) generation. Radiomics features were extracted from whole-tumor and habitat regions, selected using Z-score, correlation analysis, recursive feature elimination with cross-validation (RFECV); and Least Absolute Shrinkage and Selection Operator (LASSO). Machine learning models were trained and evaluated with receiver operating characteristic (ROC), calibration, decision, and confusion matrix analyses.

This retrospective study was approved by the Ethics Committee of Tongren People’s Hospital on May 23, 2024. Written informed consent was waived due to the retrospective nature of the study and use of de-identified imaging data. The overall study workflow is depicted in **Figure 1**. All procedures were conducted in accordance with the ethical standards of the institutional research committee and the Declaration of Helsinki.

### 2.2 Manual segmentation and image preprocessing

Tumor regions of interest (ROIs) were manually segmented layer by layer on T2WI sequences by two physicians with more than five years of experience in obstetric and gynecologic imaging diagnosis, using ITK-SNAP software (University of Pennsylvania Image Computing and Science Laboratory, version 3.6.0). Thin-slice CE-T1WI images were used as for reference during the segmentation process. In addition, to ensure the accuracy of the segmentations, all ROIs were reviewed by a senior radiologist with over fifteen years of experience, and the best segmentation was selected for subsequent analysis. Although formal reproducibility testing was not conducted in the present study, inter-observer variability was minimized through dual independent segmentation, senior expert review, and adherence to standardized delineation criteria.

Following segmentation, N4 bias field correction was applied to all images to eliminate common intensity inhomogeneities. An adaptive normalization technique was then used for each image to remove extreme voxels with intensity values above the 99th percentile or below the 1st percentile, reducing the influence of outliers on downstream analysis. Finally, all image intensities were normalized to a range of 0 to 1 using min-max normalization to ensure data consistency and comparability. All normalization procedures were performed independently within each training cohort, and the parameters derived from the training data were applied to the corresponding testing cohort to avoid data leakage.

### 2.3 Tumor habitat subregion segmentation and quantitation

**Figure 2:**
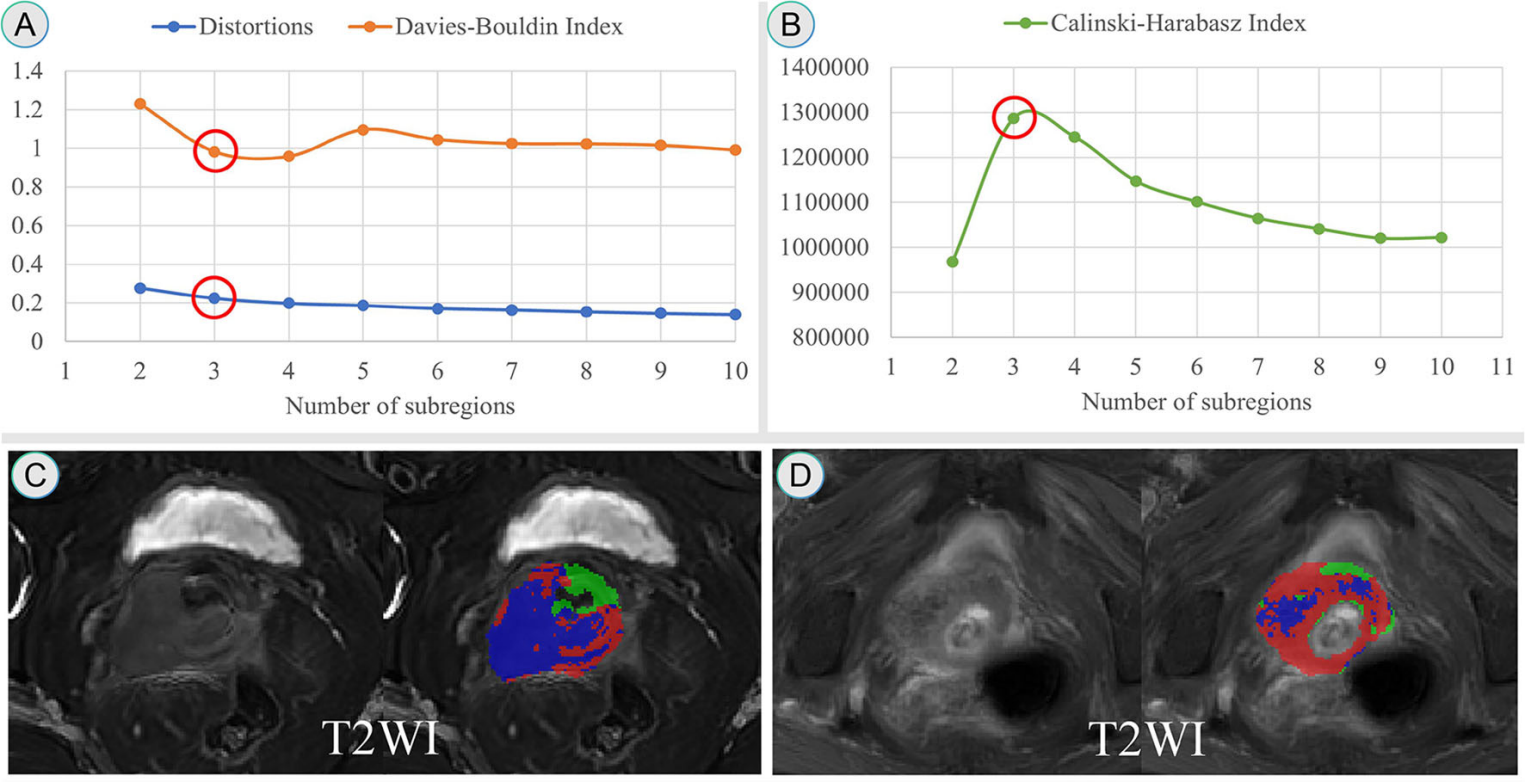
Determination of the optimal number of tumor habitat subregions and visualization on T2-weighted MRI images. **(A, B)** Clustering performance evaluated by distortion, Davies–Bouldin, and Calinski–Harabasz indices identified three optimal subregions (red circles). **(C, D)** Representative PMI-negative **(C)** and PMI-positive **(D)** cases showing overlaid habitat maps, with red, green, and blue representing habitats 1–3, respectively.

All pixel intensity values within the ROI on the T2WI sequence for all patients in the training set were extracted. The K-means unsupervised clustering algorithm was applied, with the number of clusters sequentially set from 2 to 10, and the results were statistically analyzed for each cluster number. Based on three quantitative metrics—distortions, Davies-Bouldin Index, and Calinski-Harabasz Index (19–23) —the optimal cluster number was determined at the elbow point, which corresponded to the smallest Davies–Bouldin Index and the largest Calinski–Harabasz Index. The tumor region was then divided into distinct subregions accordingly. As shown in **Figures 2A, B**, the optimal number of habitats was three. Each subregion exhibited different signal characteristics that may reflect distinct tissue components: Habitat 1 corresponded to areas with high T2WI signal intensity, suggesting higher water content or edema; Habitat 2 showed intermediate T2WI signal intensity, representing viable tumor tissue; and Habitat 3 exhibited low T2WI signal intensity, indicating more solid tumor components with higher cellular density. **Figures 2C, D** show representative MRI images of PMI-positive and PMI-negative cases, respectively.

### 2.4 Features extraction

This study was conducted on the u-AI Research Portal (United Imaging Intelligence, China, Version: 20240430), which is developed using the Python programming language (version 3.7.3, https://www.python.org) and integrates the widely adopted PyRadiomics package (https://pyradiomics.readthedocs.io/en/latest/index.html). We extracted 14 shape features, 18 first-order statistical features describing the intensity distribution of voxels within the ROI, and 72 texture features characterizing the gray-level spatial relationships within the whole-tumor ROI. To further enrich the feature set, 24 different image filtering techniques were applied to the original images, including but not limited to mean filtering, Gaussian filtering, wavelet transformation, and logarithmic filtering. Subsequently, an additional 432 first-order statistical features and 1,728 texture features were extracted from the filtered images. A total of 2,264 radiomic features were extracted from the T2WI sequences.

In the habitat analysis, the categories of radiomic features extracted from each sub-region were consistent with those from the whole tumor. However, 14 shape features were not extracted due to the spatial discontinuity of the ROI. Additionally, because some subregions had small volumes in certain patients, 360 logarithmic filter features were excluded. Ultimately, 1,890 radiomic features were extracted from each subregion.

### 2.5 Features selection and model construction

Firstly, radiomic features were standardized using z-score normalization. Point-biserial correlation analysis, which is a special case of Pearson correlation applied to continuous features and binary outcomes, was then performed to assess the association between each feature and PMI status, and features with p-values less than 0.05 were retained. Subsequently, recursive feature elimination with cross-validation (RFECV) was applied using a support vector machine (SVM) with a linear kernel as the estimator to iteratively eliminate less informative features and identify the subset that contributed most to model performance. RFECV was implemented with 5-fold cross-validation to ensure stability and reproducibility of the selected features. To further refine the feature set, the Least Absolute Shrinkage and Selection Operator (LASSO) method was employed to select features most relevant to PMI. The optimal regularization parameter (λ) in the LASSO model was determined using 10-fold cross-validation, selecting the λ value corresponding to the minimum mean cross-validation error. All feature selection and model training procedures were performed within the training cohort, and the derived parameters were applied to the testing cohort to ensure model independence and avoid data leakage.

Finally, four predictive models were constructed based on valid radiomic features from the whole-tumor and three subregions, respectively. Thirteen machine learning classifiers were evaluated to construct predictive models, including Adaptive Boosting (AdaBoost), Bagging Decision Tree (BDT), Decision Tree, Gaussian Process, Gradient Boosting Decision Tree (GBDT), K-Nearest Neighbor (KNN), Logistic Regression, Partial Least Squares Discriminant Analysis (PLSDA), Quadratic Discriminant Analysis (QDA), Random Forest, Stochastic Gradient Descent (SGD), SVM, and Extreme Gradient Boosting (XGBoost). These classifiers were selected to represent different algorithmic families, covering linear, kernel-based, ensemble, and probabilistic models. Model performance was evaluated in both the training and testing cohorts using area under the curve (AUC), accuracy, sensitivity, specificity, and F1-score, and the optimal classifier for each feature set was determined based on the highest AUC in the testing cohort.

### 2.6 Statistical analyses

The data were imported into the statistical analysis module of the uAI Research Portal software for correlation analysis. Differences in continuous and quantitative variables between patients with positive and negative PMI were assessed using either the t-test or the Mann-Whitney U test, depending on data distribution. Categorical variables and the incidence of parametrial tissue infiltration were compared using the Chi-square test or Fisher’s exact test as appropriate. A p-value of less than 0.05 was considered statistically significant. The diagnostic performance of the models was evaluated through receiver operating characteristic (ROC) curve analysis. The AUCs were compared descriptively, and additional evaluations were performed using the net reclassification improvement (NRI) and integrated discrimination improvement (IDI) metrics.

## 3 Results

### 3.1 Characteristics of patients

A total of 110 patients aged 38–68 years (median age, 52 years) were included in this study. Among them, 60 patients were pathologically confirmed as PMI-positive and 50 were PMI-negative. All patients were clinically staged as FIGO IB–IIA before treatment, and 16 PMI-positive patients were upstaged to FIGO stage IIB based on postoperative pathological confirmation. Patients were randomly assigned to the training and testing cohorts at a ratio of 8:2, resulting in 88 patients in the training cohort and 22 patients in the testing cohort. The general clinical characteristics of patients are summarized in **Table 1**. In the training cohort, a significant difference was observed in lymphovascular space invasion (LVSI) status between the PMI-positive and PMI-negative groups (p < 0.001). No significant differences were found in age, tumor size, pathological type, or degree of differentiation between the two cohorts (all p > 0.05).

**Table 1:** The general clinical information of patients.

Characteristics		Training cohort(n=88)			Testing cohort(n=22)		
		PMI-positive (n=48)	PMI-negative (n=40)	*p*	PMI-positive (n=12)	PMI-negative (n=10)	*p*
Age		56.52 ± 9.71	54.05 ± 10.04	0.245	52.92 ± 10.03	57.80 ± 8.97	0.247
Tumor size (mm)		32.44 ± 13.95	31.75 ± 12.88	0.812	33.00 ± 11.60	30.60 ± 6.33	0.566
FIGO staging				0.505			0.852
	I	23 (47.92)	15 (37.50)		5 (41.67)	3 (30.00)	
	IIA	17 (35.41)	19 (47.50)		6 (50.00)	6 (60.00)	
	IIB	8 (16.67)	6 (15.00)		1 (8.33)	1 (10.00)	
Histological Type (%)				0.777			0.677
	SCC	41 (85.42)	35 (87.50)		8 (75.00)	8 (80.00)	
	AC	7 (14.58)	5 (12.50)		4 (25.00)	2 (20.00)	
Grade (%)				0.676			0.576
	G1	7 (14.58)	8 (20.00)		3 (25.00)	1 (10.000)	
	G2	31 (68.75)	26 (65.00)		7 (58.33)	6 (60.000)	
	G3	10 (16.67)	6 (15.00)		2 (16.67)	3 (30.000)	
LVSI (%)				<0.001			0.198
	Positive	21 (43.75)	36 (90.00)		4 (25.00)	7 (70.00)	
	Negative	27 (56.25)	4 (10.00)		8(75.00)	3 (30.00)	

AC, Squamous cell carcinoma; FIGO, International Federation of Obstetrics and Gynecology; PMI, Parametrial invasion; SCC, Squamous cell carcinoma; G1, Well differentiated; G2, Moderately differentiated; G3, Poorly differentiated; LVSI, lymph vascular space invasion.

### 3.2 Feature selection

**Figure 3:**
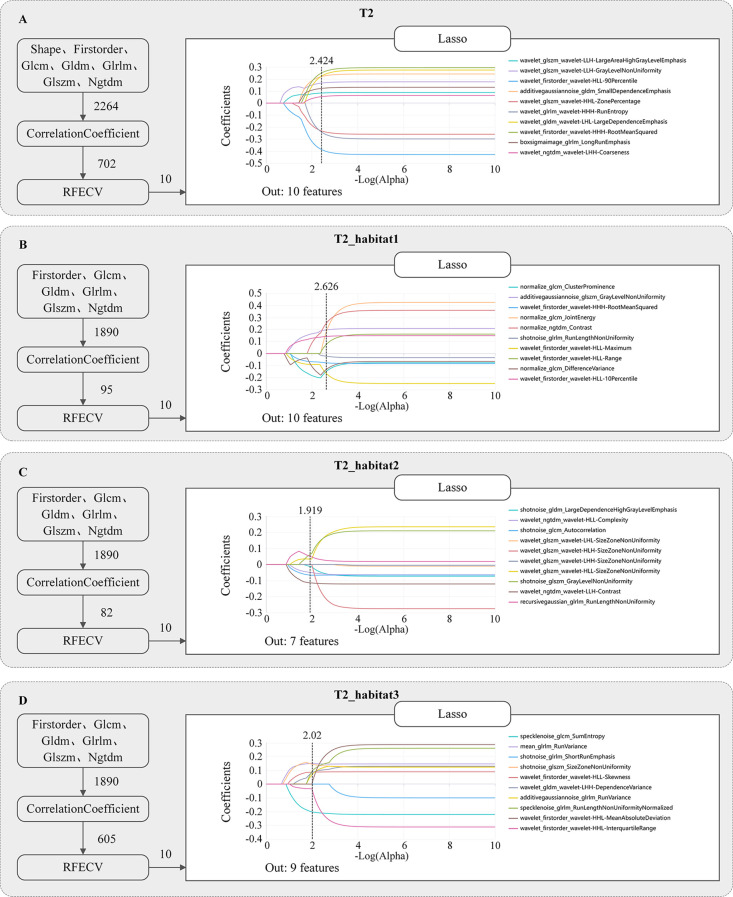
Radiomics features screened from whole tumor **(A)**, habitat 1 **(B)**, habitat 2 **(C)**, and habitat 3 **(D)**, respectively. Features were sequentially selected using correlation analysis, recursive feature elimination with cross-validation (RFECV), and the least absolute shrinkage and selection operator (LASSO) regression. The plots display feature coefficients as a function of log(α), with the vertical dashed line indicating the optimal α value corresponding to the minimum cross-validation error. The features listed on the right side of each panel represent the final retained radiomics features used for model construction.

A total of 2,264 radiomics features were extracted from the whole ROI of T2WI sequences, and 1,890 features were extracted from the ROI of each habitat. Then, the correlation coefficient method, recursive feature elimination, and LASSO regression were sequentially applied to select features (as shown in **Figure 3**). During the recursive feature elimination process, the top 10 features were retained for further analysis. Ultimately, 10, 10, 7, and 9 valid features were selected from the whole ROI, habitat 1, habitat 2, and habitat 3, respectively. The detailed features retained in each model are summarized in **Table 2**.

**Table 2:** Radiomics features retained in each model for PMI prediction.

Model	Whole ROI	Habitat 1	Habitat 2	Habitat 3
Numbers	10	10	7	9
Name.	wavelet_firstorder_wavelet-HHH-Root MeanSquared	normalize_ngtdm_Contrast	shotnoise_glszm_GrayLevelNonUniformity	mean_glrlm_RunVariance
	wavelet_gldm_wavelet-LHL-Large DependenceEmphasis	additivegaussiannoise_glszm_ GrayLevelNonUniformity	recursivegaussian_glrlm_RunLength NonUniformity	shotnoise_glszm_SizeZoneNonUniformity
	additivegaussiannoise_gldm_Small DependenceEmphasis	normalize_glcm_JointEnergy	wavelet_glszm_wavelet-HLL-SizeZone NonUniformity	specklenoise_glrlm_RunLengthNon UniformityNormalized
	wavelet_glszm_wavelet-LLH-Gray LevelNonUniformity	wavelet_firstorder_wavelet-HLL-10Percentile	shotnoise_gldm_LargeDependence HighGrayLevelEmphasis	wavelet_firstorder_wavelet-HLL-Skewness
	boxsigmaimage_glrlm_LongRunEmphasis	wavelet_firstorder_wavelet-HLL-Range	wavelet_ngtdm_wavelet-HLL-Complexity	wavelet_gldm_wavelet-LHH-DependenceVariance
	wavelet_glszm_wavelet-LLH-LargeArea HighGrayLevelEmphasis	shotnoise_glrlm_RunLengthNonUniformity	shotnoise_glcm_Autocorrelation	additivegaussiannoise_glrlm_RunVariance
	wavelet_ngtdm_wavelet-LHH-Coarseness	wavelet_firstorder_wavelet-HHH- RootMeanSquared	wavelet_ngtdm_wavelet-LLH-Contrast	wavelet_firstorder_wavelet-HHL-MeanAbsoluteDeviation
	wavelet_glszm_wavelet-HHL-ZonePercentage	normalize_glcm_DifferenceVariance		wavelet_firstorder_wavelet-HHL-InterquartileRange
	wavelet_glrlm_wavelet-HHH-RunEntropy	normalize_glcm_ClusterProminence		specklenoise_glcm_SumEntropy
	wavelet_firstorder_wavelet-HLL-90Percentile	wavelet_firstorder_wavelet-HLL-Maximum		

### 3.3 Performance of radiomics models

**Figure 4:**
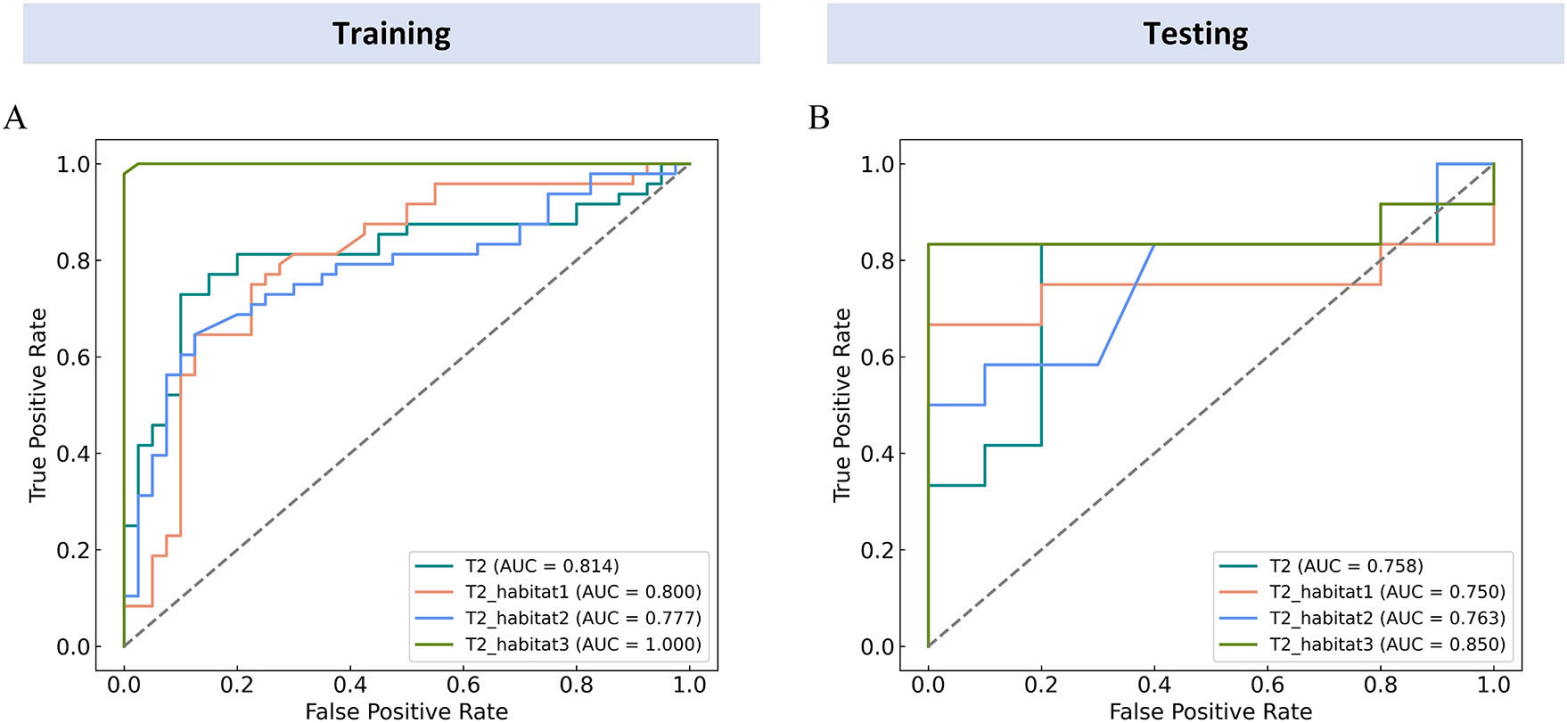
ROC curves of each model in training **(A)** and testing cohorts **(B)**. The figure shows the area under the curve (AUC) values of different models, among which the habitat 3 model achieved the highest performance with AUCs of 1.00 in the training cohort and 0.85 in the testing cohort.

The diagnostic performance of each model is summarized in **Table 3**. All models demonstrated good classification performance in distinguishing positive from negative PMI, with AUCs ranging from 0.777 to 1.000 in the training cohort and from 0.750 to 0.850 in the testing cohort. Specifically, in the training cohort, the AUCs for the whole tumor, habitat 1, habitat 2, and habitat 3 models were 0.814 (95% CI: 0.719–0.909), 0.800 (95% CI: 0.703–0.898), 0.777 (95% CI: 0.678–0.876), and 1.000 (95% CI: 0.999–1.000), respectively. In the testing cohort, the corresponding AUCs were 0.758 (95% CI: 0.535–0.981), 0.750 (95% CI: 0.511–0.989), 0.763 (95% CI: 0.554–0.971), and 0.850 (95% CI: 0.649–1.000), as shown in **Figures 4A, B**. Although the habitat 3 model achieved an AUC of 1.0 in the training cohort, 10-fold cross-validation and LASSO regularization were applied to minimize overfitting, and its performance in the testing cohort remained robust.

**Table 3:** Performances of the models in the training and testing cohorts.

Model	Method	AUC	Sensitivity	Specificity	Accuracy	Precision	F1Score
Training cohort (n = 88)							
T2(N = 10)	Quantitle_transformer>>Logistic	0.814(0.719-0.909)|	0.771	0.800	0.784	0.822	0.796
T2_habitat1(N = 10)	Quantitle_transformer>>SVM	0.800(0.703-0.898)	0.812	0.700	0.761	0.765	0.788
T2_habitat2(N = 7)	Z_score_scaler>>QDA	0.777(0.678-0.876)	0.729	0.725	0.727	0.761	0.745
T2_habitat3(N = 9)	L2_normalization>>XGBOOST	1.000(0.999-1.000)	0.979|	1.000	0.989	1.000	0.989
Testing cohort (n = 22)							
T2(N = 10)	Quantitle_transformer>>Logistic	0.758(0.535-0.981)	0.833	0.700	0.773	0.769	0.800
T2_habitat1(N = 10)	Quantitle_transformer>>SVM	0.750(0.511-0.989)	0.750	0.700	0.727	0.750	0.750
T2_habitat2(N = 7)	Z_score_scaler>>QDA	0.763(0.554-0.971)	0.833	0.600	0.727	|0.714	0.769
T2_habitat3(N = 9)	L2_normalization>>XGBOOST	0.850(0.649-1.000)	0.750	1.000	0.864	1.000	0.857

AUC, area under curve; T2, T2 weighted imaging; SVM, Support Vector Machine; QDA, Quadratic Discriminant Analysis; XGBOOST, Extreme Gradient Boosting.

**Figure 5:**
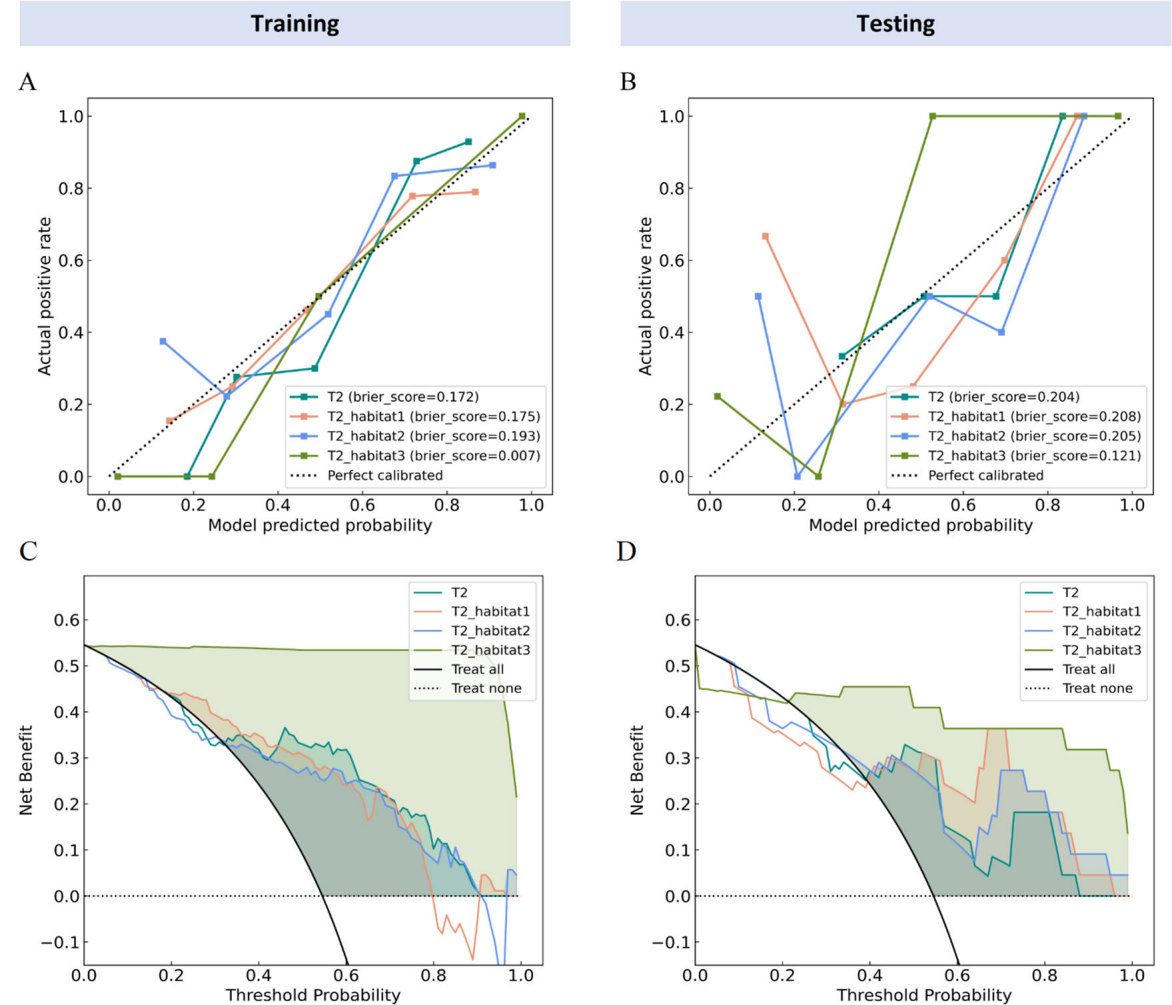
Calibration and decision curves of each model in training and testing cohorts. Calibration curves in the training **(A)** and testing **(B)** cohorts. The x-axis shows predicted probabilities, and the y-axis shows observed outcomes. The closer the curve is to the 45° line, the better the model calibration. Decision curve analyses in the training **(C)** and testing **(D)** cohorts. The x-axis represents threshold probability, and the y-axis represents net benefit. A higher net benefit indicates better clinical usefulness.

Moreover, the diagnostic accuracy of the habitat 3 model was notably superior to the whole tumor model, habitat 1 and habitat 2 models. In the training cohort, all IDI values were statistically significant, indicating significant improvement in discrimination performance (T2 vs T2_Habitat3: IDI = 0.678, P < 0.001; T2_Habitat1 vs T2_Habitat3: IDI = 0.640, P < 0.001; T2_Habitat2 vs T2_Habitat3: IDI = 0.649, P < 0.001) (**Table 4**). Similarly, in the testing cohort, the IDI values remained statistically significant, further confirming the enhanced discriminative ability of the Habitat3 model (T2 vs T2_Habitat3: IDI = 0.482, P < 0.001; T2_Habitat1 vs T2_Habitat3: IDI = 0.423, P = 0.003; T2_Habitat2 vs T2_Habitat3: IDI = 0.397, P = 0.006). The calibration curves for the habitat 3 model demonstrated good agreement over a wide range of predicted probabilities in both the training and testing cohorts (**Figures 5A, B**). The decision curve analysis demonstrated that the habitat 3 model provided a greater net clinical benefit across most threshold probabilities in both the training and testing cohorts (**Figures 5C, D**), indicating superior potential clinical usefulness for predicting parametrial invasion.

**Table 4:** Performance comparison between models in the training and testing cohorts.

Group	Old model	New model	AUC P value	NRI score	NRI P value	IDI score	IDI P value
Training cohort	T2	T2_habitat1	0.774	-0.058	0.548	0.038	0.378
	T2	T2_habitat2	0.498	-0.117	0.285	0.030	0.584
	**T2**	T2_habitat3	P<0.001	0.408	P<0.001	0.678	P<0.001
	T2_habitat1	T2_habitat2	0.730	-0.058	0.650	-0.009	0.898
	T2_habitat1	T2_habitat3	P<0.001	0.467	P<0.001	0.640	P<0.001
	T2_habitat2	T2_habitat3	P<0.001	0.525	P<0.001	0.649	P<0.001
Testing cohort	T2	T2_habitat1	0.931	-0.083	0.699	0.059	0.525
	T2	T2_habitat2	0.974	-0.100	0.690	0.085	0.478
	**T2**	T2_habitat3	0.486	0.217	0.286	0.482	P<0.001
	T2_habitat1	T2_habitat2	0.918	-0.017	0.950	0.027	0.816
	T2_habitat1	T2_habitat3	0.481	0.300	0.174	0.423	0.003
	T2_habitat2	T2_habitat3	0.519	0.317	0.132	0.397	0.006

AUC, area under curve; T2, T2 weighted imaging; NRI, Net Reclassification Improvement Index; IDI, Integrated DiscriminationImprovement Index.

## 4 Discussion

In this study, we demonstrated that radiomics models based on T2-weighted MRI of the whole tumor and its three subregions can effectively predict PMI in early-stage CC. The AUC values ranged from 0.777 to 1.000 in the training cohort and from 0.750 to 0.850 in the testing cohort. Among the four models, the Habitat 3 achieved the best performance, with AUCs of 1.000 and 0.850 in the training and testing cohorts, respectively. These findings suggest that habitat-based radiomics can serve as a noninvasive, robust, and reproducible method for preoperative PMI risk stratification.

Moreover, our findings are consistent with previous habitat-based radiomics studies. Wang et al. (18) reported similar AUC values (from 0.869 to 0.873) in their study predicting LVSI in CC, supporting the robustness of this approach. Other studies have also shown that habitat-based models outperform conventional radiomics approaches in predicting treatment response to concurrent chemoradiotherapy (4, 24). These consistent findings across studies reinforce the reliability and generalizability of habitat analysis as an imaging biomarker in CC. Compared with previous multi-sequence or multi-parametric MRI radiomics studies, our single-sequence T2-weighted MRI model combined with habitat analysis achieved comparable or even superior diagnostic performance while substantially reducing scan time and post-processing complexity. This approach also offers better cross-center reproducibility and practicality in clinical use, particularly in resource-limited settings.

Further analysis revealed that among the three habitat models, the Habitat 3 model outperformed the Habitat 1, and Habitat 2 models in predicting PMI in CC. Similar findings were reported by Wang (18) in LVSI prediction. Unlike conventional whole-tumor models that treat tumors as homogeneous entities, the habitat approach allows finer segmentation of intratumoral regions, which enables more effective characterization of microstructural and functional heterogeneity (25, 26). This spatial decomposition helps reveal imaging characteristics associated with PMI, such as local blood supply, cellular density, and restricted diffusion, thereby enhancing predictive accuracy. Furthermore, Decision curve analysis confirmed the superior net clinical benefit of the Habitat 3 model in both cohorts, highlighting its potential for individualized preoperative evaluation. Habitat 3 also exhibited more biologically meaningful spatial and textural features compared with the other habitats (27). LVSI differed significantly between PMI-positive and PMI-negative patients, suggesting its potential as a pathological indicator for preoperative risk stratification.

This study has several limitations. First, as a single-center retrospective study with a relatively small sample size, selection bias may be present. Despite strict feature selection and cross-validation, the generalizability of the results may still be limited, with a potential risk of overfitting. Second, the absence of an external validation cohort and potential differences in MRI scanners and acquisition protocols across institutions may affect the stability and reproducibility of radiomics features, thereby representing potential threats to the validity of the findings. Third, although inter-observer variability was minimized through dual segmentation and expert review, formal reproducibility testing (e.g., ICC) was not performed. In addition, the biological mechanisms underlying the superior performance of the Habitat 3 model have not been fully elucidated, and the interpretability of radiomics features at the feature level warrants further investigation. Finally, variations in patient race or geolocation may influence tumor biology, disease presentation, and clinical management of cervical cancer, which may further limit the applicability of our results to other populations. Therefore, caution is warranted when extrapolating these findings beyond the study cohort. Future prospective, multicenter studies with larger and more diverse populations are needed to further validate the robustness, generalizability, and clinical applicability of habitat-based radiomics models, and to explore their biological interpretability in greater depth.

## 5 Conclusion

In conclusion, radiomics based on habitat analysis can effectively predict the PMI status in patients with early-stage CC, with diagnostic performance superior to that of traditional whole-tumor radiomics. This approach provides a promising method for preoperative prediction of PMI in CC and lays a theoretical foundation for the deeper integration of radiomics with tumor microenvironment analysis.

## Data Availability

The raw data supporting the conclusions of this article will be made available by the authors, without undue reservation.
